# Experimental and Numerical Investigation of Effect of Static and Fatigue Loading on Behavior of Different Double Strap Adhesive Joint Configurations in Fiber Metal Laminates

**DOI:** 10.3390/ma15051840

**Published:** 2022-03-01

**Authors:** Muhammad Azeem, Muhammad Irfan, Manzar Masud, Gulfam Ul Rehman, Haider Ali, Muhammad Umair Ali, Amad Zafar, Usama Muhammad Niazi, Saifur Rahman, Stanislaw Legutko, Jana Petrů, Jiří Kratochvíl

**Affiliations:** 1 Department of Mechanical Engineering, University of Wah, Quaid Avenue, Wah Cantt 47040, Pakistan; m.azeem@wecuw.edu.pk (M.A.); gulfam.rehman@wecuw.edu.pk (G.U.R.); haider.ali@wecuw.edu.pk (H.A.); 2 Electrical Engineering Department, College of Engineering, Najran University Saudi Arabia, Najran 61441, Saudi Arabia; miditta@nu.edu.sa (M.I.); srrahman@nu.edu.sa (S.R.); 3 Department of Mechanical Engineering, Capital University of Science and Technology (CUST), Islamabad 44000, Pakistan; manzar.masud@cust.edu.pk; 4 Department of Unmanned Vehicle Engineering, Sejong University, Seoul 05006, Korea; 5 Department of Electrical Engineering, The Ibadat International University, Islamabad 54590, Pakistan; amad.zafar@iiui.edu.pk; 6 Department of Mechanical Engineering Technology, National Skills University, Islamabad 44000, Pakistan; ukniaxi@gmail.com; 7 Faculty of Mechanical Engineering, Poznan University of Technology, 3 Piotrowo Street, 60-965 Poznan, Poland; stanislaw.legutko@put.poznan.pl; 8 Department of Machining, Assembly and Engineering Metrology, VSB Technical University of Ostrava, 17. Listopadu 2172/15 Street, 708-00 Ostrava, Czech Republic; jana.petru@vsb.cz (J.P.); jiri.kratochvil@vsb.cz (J.K.)

**Keywords:** adhesively bonded joints, double strap joint, fatigue loading, static loading, fiber metal laminate, finite element analysis

## Abstract

Double strap lap adhesive joints between metal (AA 6061-T6) and composite (carbon/epoxy) laminates were fabricated and characterized based on strength. Hand layup methods were used to fabricate double strap match lap joints and double strap mismatch lap joints. These joints were compared for their strength under static and fatigue loadings. Fracture toughness (GIIC) was measured experimentally using tensile testing and validated with numerical simulations using the cohesive zone model (CZM) in ABAQUS/Standard. Fatigue life under tension–tension fluctuating sinusoidal loading was determined experimentally. Failure loads for both joints were in close relation, whereas the fatigue life of the double strap mismatch lap joint was longer than that of the double strap match lap joint. A cohesive dominating failure pattern was identified in tensile testing. During fatigue testing, it was observed that inhomogeneity (air bubble) in adhesive plays a negative role while the long time duration between two consecutive cycle spans has a positive effect on the life of joints.

## 1. Introduction

Fiber metal laminates (FMLs) basically fall in the category of hybrid composites, consisting of alternating layers of metal alloy sheets such as aluminum and fiber-reinforced epoxy such as carbon/epoxy. FMLs were basically developed for their increased crack resistance for fatigue, and they offer substantial enhancements in stiffness, weight saving and strength against their aluminum counterparts [1]. Adhesive bonding is a method for joining solid components, especially advantageous in fiber metal laminates created by mechanical fastening [2]. In mechanical fastening, holes are drilled in a material, which itself is a crack forming process. After the application of load, cracks may propagate, leading to failure. Moreover, other advantages of FMLs over a traditional fastening are high strength-to-weight ratio, corrosion resistance and good impact and fatigue performance [3]. Different types of adhesive joints are plain butt joint, single and double lap joint, single and double strap joint, tongue and groove lap joint, joggle lap joint, etc. [4].

Double strap lap adhesive joints between metal and composite are widely used in repairing megastructures, such as aircraft, bridges, turbine blades and windmills [5]. Scientists used different techniques (hand layup, autoclaves) for the fabrication of double strap lap joints adopting various mechanical (peel ply, sandpaper, sandblast) [6,7] and chemical (sulfuric acid, phosphoric acid, chromic acid and phosphoric chromic acid anodizing) [8,9,10] surface treatments on Al alloy with differing in composite strap shape [11,12], edge geometry [13,14,15], fiber type, stacking sequence [16], overlap length [14,17,18], adhesive thickness [19] and curing temperature [20]. Tests were performed under tensile [15,21], fatigue [13,22], three [23] and five [24] point bending to measure shear strength, fracture energy of the joint and bending moment. The delamination behavior of joints was studied using finite element modeling [18,25,26,27,28], and the delaminated surface was examined under a scanning electron microscope [29].

Mechanical properties of composites are generally enhanced by the introduction of thin metallic sheets between fiber-based polymers. Generally, three important FMLs include carbon-reinforced aluminum laminate (CARALL), glass-reinforced aluminum laminate (GLARE) and aramid-reinforced aluminum laminate (ARALL) [30]. These types of FMLs exhibited high strength as compared to monolithic metal sheets [31]. Due to the increased use of such laminates in aerospace applications, their mechanical strength plays a very important role because their failure may lead to catastrophes. Therefore, researchers have tried to investigate the failure response of FMLs and explored different ways to avoid such failures. It has been concluded that the type and number of metal layers, stacking sequence and orientation of layers play an important role in the mechanical strength of FMLs [32]. It has also been observed that the quantity of adhesive influences the mechanical properties and failure response of FMLs [33].

Prediction of induced stresses and failure patterns for bonded joints is necessary for the better understanding of stress fields around the joints, as well as the damage initiation and propagation. Numerical methods provide a better and general tool for the analysis and prediction. Some numerical studies have been successfully carried out using cohesive zone modeling to predict the static and dynamic behavior of adhesively bonded joints [34,35,36]. In these studies, the damage mechanism was based on progressive damage modeling as well as the cohesive zone method (CZM). The use of finite element methods (FEMs) to analyze adhesively connected joints with composite adherends has facilitated researchers in understanding the structures and their failure mechanisms [37,38].

A comprehensive study has been reported in the literature on the double strap match lap joints either through experimentation or computational techniques. The work involving the study of mismatch double strap lab joints has not been reported in the literature. Therefore, this research work involves the fabrication and experimental tensile and fatigue testing of double strap lap adhesive joint (match and mismatch) between metal and composite (aluminum alloy 6061-T6 metal with carbon/epoxy composite laminates) and validation with simulation results. Two new approaches are explored in this research. One is related to the carbon fiber orientation (woven) in carbon/epoxy laminates while the other is related to the comparison between double strap (match and mismatch) geometry configuration. These configurations were fabricated using the hand layup technique, which is quite economical, rather than manufacturing through autoclaves. Failure load was determined using tensile test. Mode II fracture energy values (G_IIC_) were determined analytically using experimental values, and then numerical simulation of the tensile test was carried out using ABAQUS/Standard software using cohesive zone modeling. Fatigue tests determined the cyclic life of both configurations. Finally, the fracture surface between aluminum and carbon/epoxy laminates was observed using an optical microscope.

## 2. Experimental Procedures

### 2.1. Materials

A plate made of aluminum alloy 6061-T6 (Maxtech-Me, Sharjah, UAE), having a thickness of 5 mm, was used in this study. This alloy is widely used in the aerospace and automobile industries, especially for the manufacturing of aircraft wings and fuselages [39]. The plasticity of the alloy plays an important role in determining the fracture energy of the FMLs. The alloy AA6061-T6 provides moderate strength with very good formability. The chemical composition of this alloy is given in Table 1.

Plain-woven carbon fabric, which is used as a strap material [41], was used to make carbon-reinforced aluminum laminate (CARALL) composites. It has high tensile strength and continuous fiber with 3000 filament tows in the present case [42]. It is a reinforcement material with a high modulus of elasticity and high stiffness in tension and compression. It has good fatigue performance. It is used in prepreg, weaving, braiding, filament winding and also in different aerospace applications.

For bonding of two constituents, the adhesive system used consists of Araldite LY5052 epoxy resin (Tei Composites, Chang Hua, Taiwan) and Aradur 5052 hardener (Tei Composites, Chang Hua, Taiwan) [43]. It is a low viscosity epoxy resin used for the hand layup technique. The curing time for this is 48 h at room temperature. After curing, the resin should be postcured at 100 °C for 4 h in order to optimize the extent of cross-linkage and to enhance composite properties. This epoxy is not particularly optimized for adhesion to aluminum alloy. Surface preparation is necessary to obtain good bonding between epoxy and Al sheets [44,45,46].

### 2.2. Surface Preparation

The surface of the aluminum alloy was prepared using two processes. In the first process, the surface of the Al alloy was ground with different sandpapers having grit sizes of 180, 320, 600, 1000, 1200 and 2000. The ground surface was then degreased using 11% NaOH solution for 15 min followed by deoxidation using a solution containing 10% Na_2_Cr_2_O7 with 30% H_2_SO_4_ in water for 15 min.

Subsequently, the plates were anodized according to the American Society of Testing Materials (ASTM) standard D3933-98 using a solution of 12% H_3_PO_4_ at 12 V DC for 25 min each. The resin layup was done immediately afterward [29].

### 2.3. Resin Layup Process

**Figure 1:**
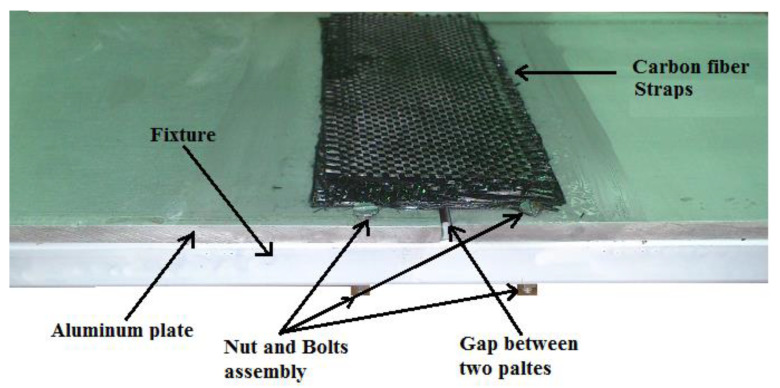
Bonding between carbon fiber straps and aluminum plates.

The resin layup process was carried out using the conventional hand layup technique. It can be seen that the hand layup process cannot be carried out without assembling the substrate (aluminum) on a fixture, especially in the case of the double strap joint, as shown in Figure 1.

The total thickness of the reinforced (carbon fiber) strap required, as per the ASTM standard D 3528 [47], was 2.5 mm on both sides of aluminum plates. The thickness of each layer of woven carbon fiber strap was 0.28 mm, as measured using a micrometer dial gage. Therefore, to obtain the total thickness of 2.5 mm, nine layers of fiber were stacked on each side of Al plates. All the assembled plates were postcured for 4 h at 100 °C to improve the mechanical properties of the joint. So, four sets of specimens were prepared for tensile and fatigue testing. These specimens were given certain identification codes as given in Table 2.

### 2.4. Specimen Configuration

**Figure 2:**
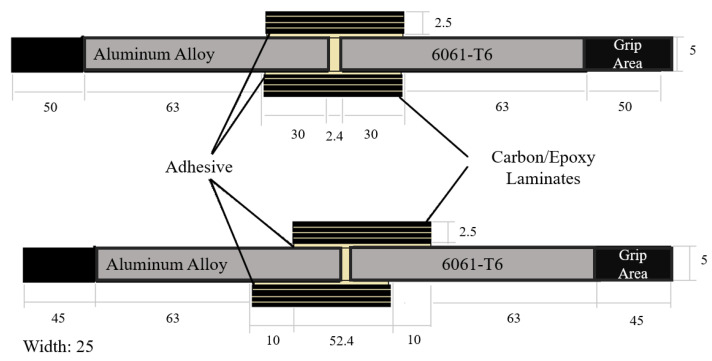
Double strap match and mismatch lap joint between aluminum and carbon fibers (all the dimensions are in millimeters).

The specimens were prepared according to the ASTM standard D 3528-96 [47] as shown in Figure 2.

**Figure 3:**
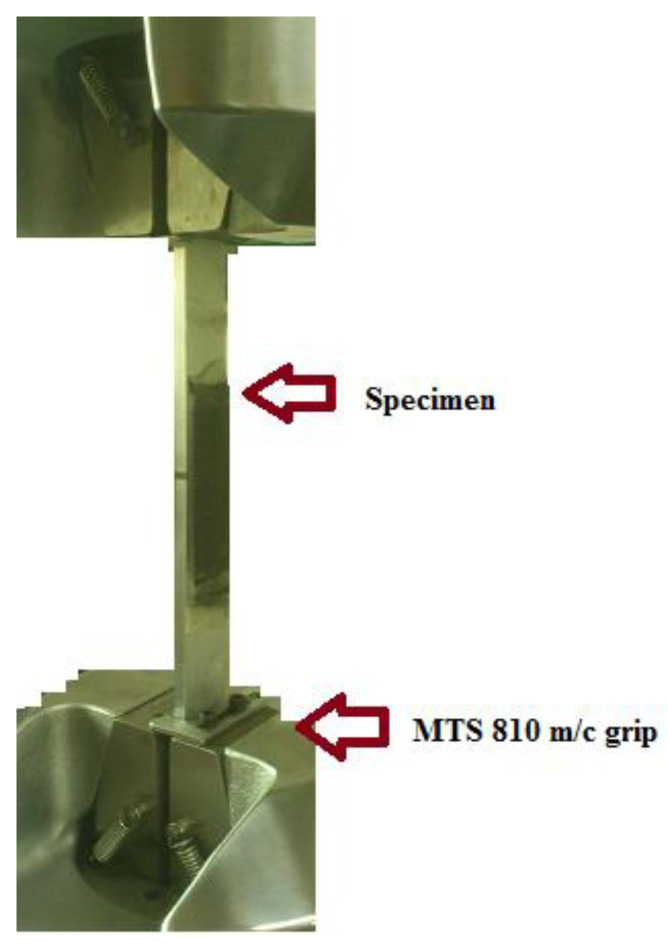
Specimen under tensile test.

Standard tensile tests, as shown in Figure 3, were carried out, and load–displacement curves were obtained; along with these curves, G_IIC_ values were calculated using analytical expression [48].

Fracture energy G_IIC_ was calculated for both sets of tensile specimens. The formulation used for fracture energy calculation is given in Equation (1).

(1) $$G_{\mathrm{IIC}}=\frac{F_{\max}^{2}\left(E_{b}t_{b}+3E_{s}t_{s}\right)}{3E_{b}t_{b}w^{2}\left(E_{b}t_{b}+E_{s}t_{s}\right)}$$

where:

G_IIC_: fracture energy;

F_max_: maximum bearing force;

E_b_: Young’s modulus of base metal (aluminum);

E_s_: Young’s modulus of overlap strap (carbon/epoxy);

t_b_: thickness of base metal (aluminum);

t_s_: thickness of overlap strap (carbon/epoxy);

w: width of the specimen.

## 3. Characterizing Method and Results

### 3.1. Tensile Test

**Figure 4:**
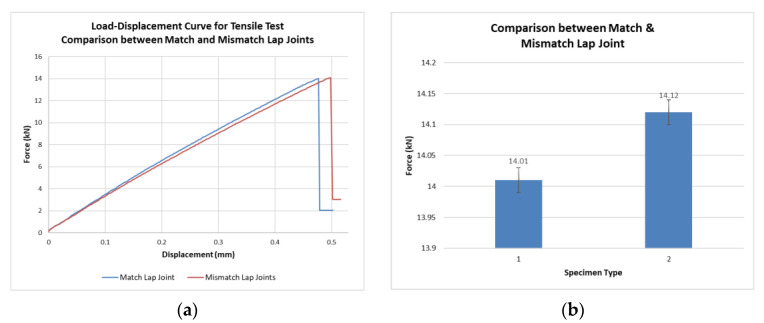
Comparison between match and mismatch lap joints: (**a**) load–displacement curve for tensile test; (**b**) failure loads along with error bars.

In order to determine the effect of the match and mismatch straps on the strength of the double strap lap joint, all specimens were tested up until failure in tension, using ASTM standard D 1002-01 [49]. The experiments were conducted using a hydraulic MTS 810 machine under crosshead rate of 1.27 mm/min, and the distance from the end of the lap to the jaws was 63 mm. During the tests, displacement and the loading histories were obtained from the load cell embedded on the loading fixture using a data acquisition system. Three specimens were tested in both cases. Mean values are shown below in Table 3. Figure 4 shows the typical load–displacement curve and bar chart of the double strap match and mismatch lap joints. The strength of double strap lap adhesive joints is almost the same in both cases.

**Figure 5:**
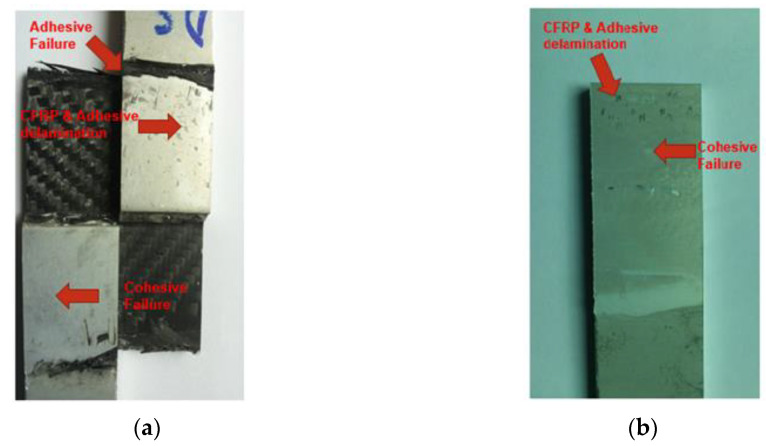
Fracture surfaces of double strap lap joints (**a**) match (**b**) mismatch.

The fracture surfaces of the match and mismatch double strap lap joints are shown in Figure 5. In the match-type joint, there is a combination of adhesive and cohesive failure on both sides of the aluminum sheets, whereas only cohesive failure was observed in the mismatch-type joint but with higher failure loads. As the joints were prepared using the hand layup method, there was a possibility of shrinkage of adhesive between the layers of the CFRP during the postcuring process. This shrinkage in adhesive became a reason for the crack initiation from the layers of the CFRP. The crack growth was tracked from the CFRP to the adhesive, filled between the CFRP and aluminum sheets until failure of the joints. Delamination of CFRP and adhesive on the aluminum sheets was also observed in both joint types.

### 3.2. Fatigue Test

In addition to the joint strength, the fatigue performance of match and mismatch strap joints under cyclic loading was also examined using ASTM standard D 3166-99 [50]. The fatigue tests were conducted on the hydraulic MTS 810 machine under a frequency of 30 cycles/s (30 Hz). Tensile–tensile fluctuating sinusoidal loading with a load ratio σ_min_/σ_max_ = 0.1 was applied on the specimens. The maximum load Pmax in the fatigue tests was set at 50% of the average failure load or yield strength, and the minimum load was set to approximately 10% of the maximum load (Pmax). Therefore, the obtained values were 6 and 0.6 kN, respectively. Both match and mismatch strap samples were tested under the same cyclic loading conditions, and the results are compiled in Table 4.

**Figure 6:**
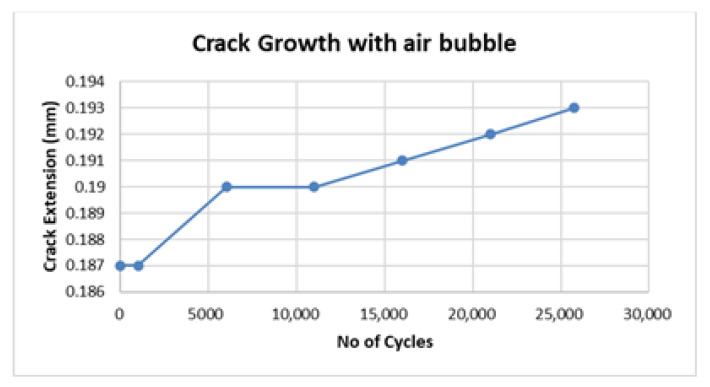
Crack growth due to inhomogeneity.

Match and mismatch joints were compared through analysis of crack growth with respect to the number of applied cycles in fatigue testing. From Table 4, it is evident that two samples from specimen 3 failed at close to 80,000 cycles whereas all three samples from specimen 4 failed at close to 110,000 cycles. However, there was one anomaly in sample 1 from specimen 3, which failed at 25,737 cycles, a markedly lower number than those of other samples from the same specimen. To further analyze this anomalous behavior, a graph between number of cycles and crack extension is shown in Figure 6. The graph shows crack initiation and growth from 1000–6000 cycles and 11,000–25,737 (failure) cycles, whereas the adhesive restricted the crack initiation for 0–1000 cycles and crack growth for 6000–11,000 cycles.

**Figure 7:**
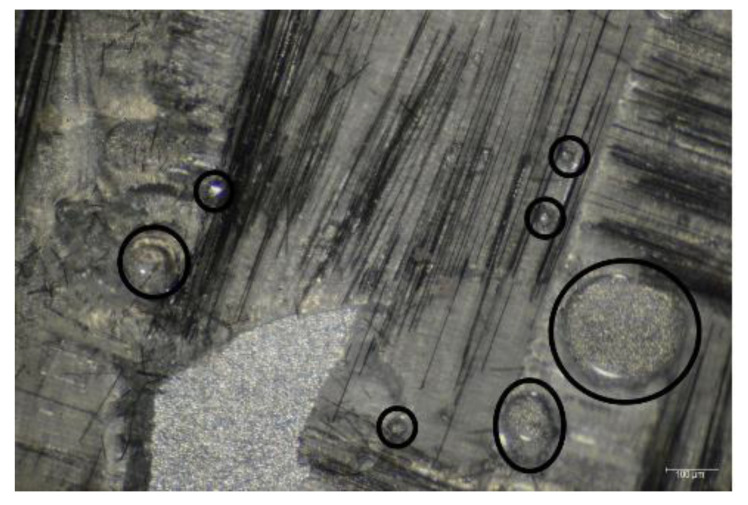
Carbon/epoxy delamination with air bubbles.

Fractography using an optical microscope revealed that air bubbles were trapped within adhesive during fabrication. The presence of these air bubbles decreased the bond strength. In fractography images shown in Figure 7, circled areas show the presence of air bubbles.

**Figure 8:**
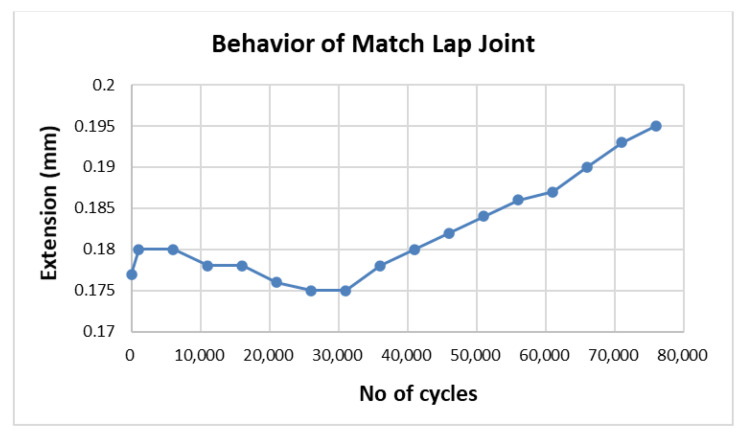
Fatigue crack growth in double strap match lap joint (specimen 3, sample 2).

**Figure 9:**
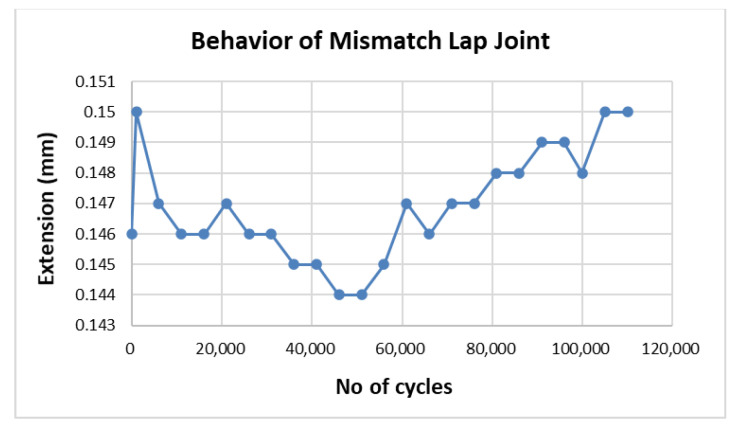
Fatigue crack growth in double strap mismatch lap joint (specimen 4, sample 1).

Similar behavior was observed in samples 2 and 3 of specimen 3, and the behavior of sample 2 is shown in Figure 8. The three samples of specimen 4 exhibited the same behavior; hence, only one is presented in Figure 9.

In both Figure 8 and Figure 9, there is a dip, showing crack contraction due to strain hardening, in the graphs after the first initiation of the crack. A small amount of strain hardening was observed in the match lap joint as compared to the mismatch lap joint. After the contraction and going to a minimum dimension of 0.175 mm in the match lap joint and 0.144 mm in the mismatch lap joint, the crack extended.

**Figure 10:**
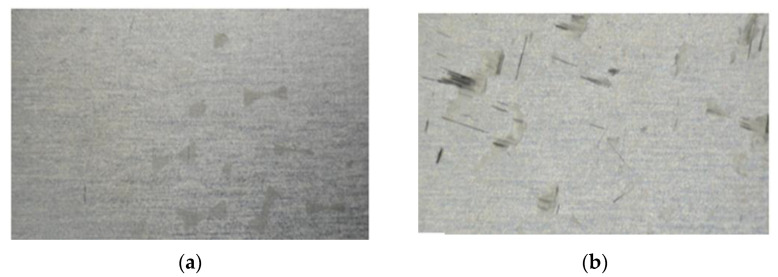
Carbon/epoxy delamination: (**a**) double strap match lap joint; (**b**) double strap mismatch lap joint.

Fractography using an optical microscope revealed that the adhesion between aluminum and carbon/epoxy laminates is stronger in double strap mismatch lap joint as compared to match lap joint. Figure 10a shows the double strap match lap joint, while Figure 10b shows the double strap mismatch lap joint.

Strain hardening is strongly related to the time interval between the consecutive cycles. As the time interval between consecutive cycle spans was increased, it increased the strain hardening and hence increased the fatigue life of the specimen.

**Figure 11:**
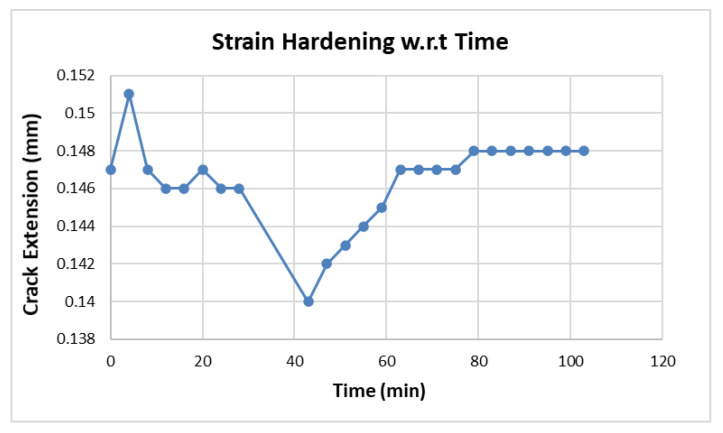
Strain hardening phenomenon with respect to time.

In Figure 11, each crack extension point is plotted after a time interval of 4 min, but during 31,000–36,000 cycles, the time interval was increased to 15 min. This gave a contraction in crack by 4.2% from 0.146 to 0.14 due to strain hardening.

## 4. Finite Element Analysis

**Figure 12:**
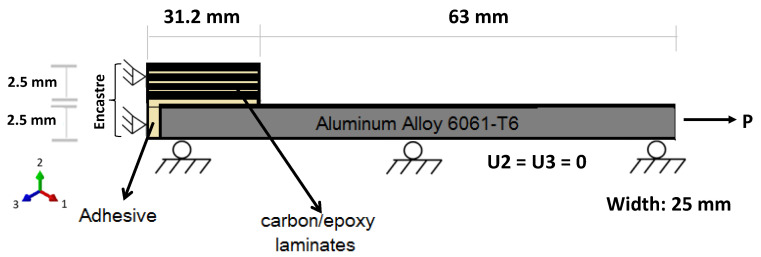
Schematic representation of quarter specimen with boundary conditions.

In addition to the experiments, the performance of adhesive in double strap lap joint was determined using finite element analysis (FEA). The analysis was conducted using commercial software ABAQUS based on the geometric configuration of the specimens described earlier in Figure 2; only quarter geometry was modeled because of symmetry, as shown in Figure 12. All dimensions are in millimeters.

Parts were modeled according to dimensions using a 2D planer deformable shell. Isotropic materials were used for substrate (aluminum) and overlap strap (carbon fiber) using values of density, elastic and plastic constants for simulating the behavior of the material under mechanical loading. The adhesive layer properties before fracture were assigned as traction in the form of stiffness in normal (E/K_nn_), first (G_1_/K_ss_) and second directions (G_2_/K_tt_). Damage properties were assigned using damage for traction separation law in the form of maximum stress damage, G_IIC_ value was determined using an analytical solution with the help of experimental results as shown above in Equation (1). The obtained values for aluminum, carbon fiber and the cohesive element are given in Table 5 and Table 6.

An assembly module was used for assigning material to the specific part and converting the assembly from a local coordinate system to a global coordinate system. Interaction was defined using a tie constrained between adherent, attachment and adhesive. Boundary conditions were applied on the assembly as described below in Figure 12. Attachment and adhesive were constrained in x-, y- and z-directions and adherent were free to move in the x-axis and restricted in the other two directions.

**Figure 13:**
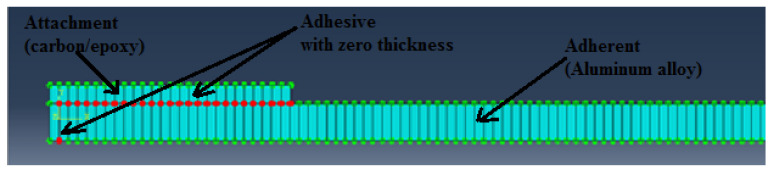
Meshed specimen with zero thickness of cohesive elements.

The model was independently meshed with respect to parts. Aluminum and carbon epoxy laminates were meshed using a structured quadratic plane stress element referred to in ABAQUS as a CPS4R element. The adhesive was meshed using a sweep quadratic (where sweep path was in the upward direction) cohesive element referred to in ABAQUS as COH2D4 through element deletion [51]. Mesh sensitivity analysis was performed, and it was established that the final mesh was to be a compromise between the output quality and time required. Five different cases were investigated with the number of elements of 373, 481, 522, 637 and 759. The mesh convergence study shows that there is no major difference in the results as the number of elements is increased; therefore, the case with the number of elements of 522 was chosen for the study. After part meshing, the mesh was edited and zero thickness was assigned for the adhesive part as shown in Figure 13.

**Figure 14:**
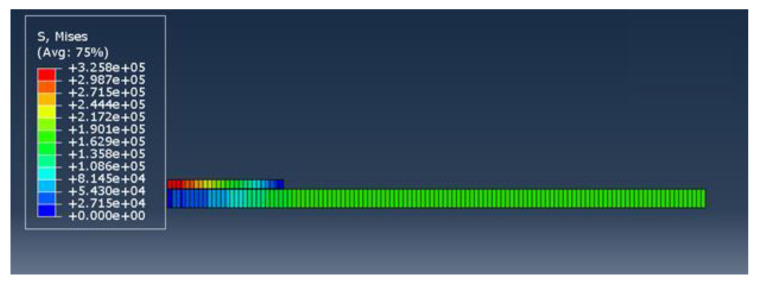
Final shape of the model after loading.

The job was created and submitted after setting field outputs. ABAQUS determined principal stresses, strains and nodal displacements within the bond. Selected field outputs were visualized using the visualization module as shown below in Figure 14. The nodal values of these quantities were exported into Excel for more processing.

**Figure 15:**
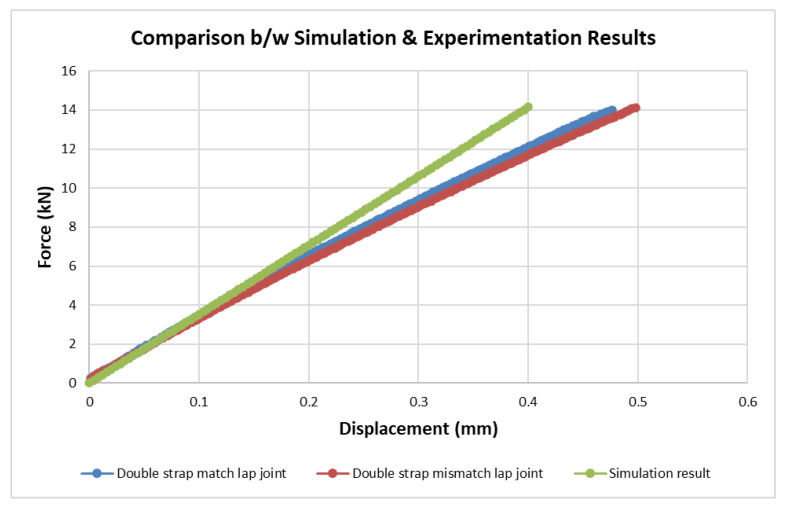
Comparison between simulation and experimental results.

Computational analysis was carried out on the double strap lap joints and is presented in Table 7. The response of the match and mismatch joints under tensile loading depends on fracture energy, the value of which is almost the same for both configurations; therefore, given results are compared with the match joint only. However, a comparison with the mismatch joint is also presented in Figure 15. The failure load and fracture energy in simulation and experimentation are almost equal with only 0.5% and 0.58% relative error, respectively.

## 5. Conclusions

This study focuses on the relationship between the strength of a double strap joint and the novel orientation (mismatch) of plain-woven carbon fiber straps on an aluminum 6061-T6 plate and the comparison with the strength of a traditional double strap match lap joint. Both tensile and fatigue strengths were evaluated under the influence of preferable surface treatment and manufacturing technique and validated through computational technique. Fractography was used to observe possible failure mechanisms.

The tensile testing of both double strap match lap joint and double strap mismatch lap joint showed similar behavior with adhesive and cohesive failures. However, the mismatch-type joint has higher strength by 0.8%, increased ability to extend by 4.3% and improved fracture energy by 1.6% when compared to the match-type joint. In the fatigue test, the double strap mismatch lap joint showed a 45% increase in fatigue life (failure at ~110,000 cycles) compared to the double strap match lap joint (failure at ~76,000 cycles). In fatigue, cohesive failure was observed in both joints. A phenomenon of crack contraction, possibly due to the strain hardening in the adhesive, was observed. The crack contraction increases with the increase in the rest time between two consecutive cycle spans by 4.2%. Inhomogeneities such as air bubbles, dust or oil particles in the adhesive decreased the strength of the joint, making it fail at one-third of the number of cycles when compared to inhomogeneity-free joints.

The experimental results were validated with simulation results from ABAQUS/Standard software using 522 elements. The experimental and simulated G_IIC_ values were in close proximity with only 0.5% relative error when compared as a function of the load–displacement curve. The failure pattern in the form of traction–separation law was in bilinear or triangular form.

## Figures and Tables

**Table 1:** Chemical standard composition of Al 6061-T6 alloy plate [40].

**Element**	Si	Mg	Cu	Cr	Fe	Mn	Zn	Ti	Al
**Weight % (max)**	0.8	1.2	0.4	0.35	0.7	0.15	0.25	0.15	Bal

**Table 2:** Codes assigned to different specimens prepared.

Specimen No.	Specimen Configuration	Code Assigned
1	Tensile test for double strap match lap joint	TT-M
2	Tensile test for double strap mismatch lap joint	TT-MM
3	Fatigue test for double strap match lap joint	FT-M
4	Fatigue test for double strap mismatch lap joint	FT-MM

**Table 3:** Summary of the tensile test results.

Specimen Type	Failure Load kN	Failure Load Standard Deviation kN	Extension mm	Extension Standard Deviation mm	Fracture Energy (G_IIC_) J/m^2^
1	14.01	0.01	0.47	0.003	402
2	14.12	0.02	0.49	0.005	408.34

**Table 4:** Summary of the fatigue test results.

Specimen Type	No. of Cycles		
	Sample 1	Sample 2	Sample 3
3	25,737	76,135	76,243
4	110,000	110,340	110,169

**Table 5:** Isotropic properties of aluminum and carbon fiber.

Materials	Density g/cm^3^	Tensile Modulus GPa	Tensile Strength MPa	Poisson’s Ratio
Aluminum plate	2.7	68.9	241	0.33
Carbon fiber	1.8	230	3450	0.35

**Table 6:** Directional stiffness properties of adhesive.

E/K_nn_ GPa	G1/K_ss_ GPa	G2/K_tt_ GPa
3.44	1.27	1.27

**Table 7:** Simulation result of tensile test.

Simulation Result	Failure Load kN	Extension mm	Fracture Energy (G_IIC_) J/m^2^
Double strap lap joint	13.97	0.39	399.71

## Data Availability

All the data is available within the manuscript.

## Nomenclature

FMLs	Fiber metal laminates
CZM	Cohesive zone model
CARALL	Carbon-reinforced aluminum laminate
ASTM	American Society of Testing Materials
FEA	Finite element analysis
